# A new statistical approach to combining p-values using gamma distribution and its application to genome-wide association study

**DOI:** 10.1186/1471-2105-15-S17-S3

**Published:** 2014-12-16

**Authors:** Zhongxue Chen, William Yang, Qingzhong Liu, Jack Y Yang, Jing Li, Mary Qu Yang

**Affiliations:** 1 Department of Epidemiology and Biostatistics, Indiana University School of Public Health, 1025 E. 7th street, PH C104, Bloomington, Indiana, 47405, USA; 2 Department of Computer Science, George W. Donaghey College of Engineering and Information Technology, University of Arkansas at Little Rock, 2801 S. University Avenue, Little Rock, Arkansas 72204, USA; 3 Department of Computer Science, Sam Houston State University, Huntsville, Texas 77341, USA; 4 Center for Compuational Biology and Bioinformatics, Indiana University School of Medicine, Indianaplois, Indiana, 46202, USA; 5 Division of Biostatistics and Biomathematics, Massachusetts General Hospital and Harvard Medicial School, Boston, Massachusetts 02114, USA; 6 MidSouth Bioinformatics Center, Department of Information Science, George W. Donaghey College of Engineering and Information Technology, University of Arkansas at Little Rock, 2801 S. University Avenue, Little Rock, Arkansas, 72204, USA; 7 Joint Bioinformatics Graduate Program, University of Arkansas at Little Rock and University of Arkansas for Medical Sciences, Little Rock, Arkansas 72204, USA

**Keywords:** Fisher test, Lancaster method, rare variant association test, z-test

## Abstract

**Background:**

Combining information from different studies is an important and useful practice in bioinformatics, including genome-wide association study, rare variant data analysis and other set-based analyses. Many statistical methods have been proposed to combine p-values from independent studies. However, it is known that there is no uniformly most powerful test under all conditions; therefore, finding a powerful test in specific situation is important and desirable.

**Results:**

In this paper, we propose a new statistical approach to combining p-values based on gamma distribution, which uses the inverse of the p-value as the shape parameter in the gamma distribution.

**Conclusions:**

Simulation study and real data application demonstrate that the proposed method has good performance under some situations.

## Background

To combine information from individual studies, many statistical approaches have been proposed. For example, meta-analysis with fixed or random effects has been intensively used to combine information from separate relevant genome-wide association studies (GWASs). However, in practice sometimes it may not be able to get all the statistics that we need, such as odds ratio and its 95% confidence interval; instead, only p-value from each study is available. In this case, combining p-values from independent studies should be used. In the literature, many statistical methods have been proposed to combine p-values [1-14]. For example, it has been shown that the Fisher test is more robust than the z-test and is commonly used for genetic data [15-26]. On the other hand, if the effects have the same direction and/or similar sizes, z-test is more powerful than the Fisher test. Some studies have shown that the weighted z-tests with weight equals to the sample size or the inverse of the standard error may perform better than the unweighted z-test under certain situations [27]. However, it has also been shown that there is no uniformly most powerful method [1]. Therefore, it is desirable to find a test which is more powerful than others for given situations. For instance, in GWAS meta-analysis, it is very common that the genetic effects of the same single-nucleotide polymorphism (SNP) from different studies are heterogeneous due to various environmental factors and study populations. Therefore, the fixed effect model cannot be applied and a p-value combining method is preferred.

Lancaster generalized Fisher test by giving certain degrees of freedom to individual studies when combine p-values based on the chi-square distribution. When the degrees of freedom (df) equal to two for each study, Lancaster's test is identical to the Fisher test. Recently, Chen and Nadarajah have studied another special case of Lancaster's test where the df is one for each study [14]. They have shown that their test can also be viewed as a weighted z-test with the "weight" equals to the estimated effect, defined as the estimated mean difference divided by the estimated standard error, which can be calculated by $\left|{\text{Φ}}^{-1}\left(p_{i}\right)\right|$, where *p_i _*is the one-sided p-value from the i^th ^study and ${\text{Φ}}^{-1}\left(.\right)$ is the inverse of the cumulative density function (CDF) of the standard normal distribution, N(0,1).

Methods based on the gamma distribution (GDM) are also available in the literature. In fact, Lancaster's methods are special cases of GDMs. GDMs are more flexible and potentially can be more powerful in some situations when appropriate parameters (e.g., the shape parameter in the gamma distribution) are chosen. However, it is usually difficult to set appropriate parameters before we see the data. In this paper, we propose a GDM, which adaptively chooses the shape parameter of the gamma distribution for each individual study. We compare the performance of the proposed test with existing methods through simulation studies. We also use real data application to illustrate the use of the new approach.

## Methods

Suppose we have K independent studies and their associated p-values p_i _(i = 1,2,...,K). Under the null hypothesis that there is no effect for all studies, the p-values from individual studies are uniformly distributed between 0 and 1. The weighted z-tests are formulated as follows:

(1) $$Z_{W}=\overset{k}{\underset{i=1}{\sum }}w_{i}{\text{Φ}}^{-1}\left(p_{i}\right)/\sqrt{\overset{k}{\underset{j=1}{\sum }}w_{j}^{2}}.$$

where *w_i _*is the weight for study *i*. When all *w_i _*= 1, the above test is the unweighted z-test, also called the Stouffer test [5]. When w_i _= n_i_, where n_i _is the sample size for study *i *, it is called the Mosteller-Bush test [8]. Other researchers suggested the use of the square root of the sample size $\sqrt{n_{i}}$ or the inverse of the estimated standard error $1/\hat{se}$ as weight [27].

Other ways to combine p-values are based on the following property: if $Y_{1},Y_{2},\ldots ,Y_{K}$ are *K *independent random variables and each has a chi-square distribution with df equal to *d_i_*, then their sum has a chi-square distribution with df equal to the sum of their df's. Fisher [9] found that if K random variables $X_{1},X_{2},\ldots ,X_{K}$ are independent and identically uniformly distributed between 0 and 1, then each $-2\text{log}\left(X_{i}\right)$ has a chi-square distribution with df = 2 and their sum $-2\overset{k}{\underset{i=1}{\sum }}\text{log}\left(X_{i}\right)$ has a chi-square distribution with 2K df, $\chi^{2}\left(df=2k\right)$. Based on this fact, Fisher used test statistic $-2\overset{k}{\underset{i=1}{\sum }}\text{log}\left(P_{i}\right)$ and compared it to $\chi^{2}\left(df=2k\right)$ to calculate the overall p-value. Lancaster [3] generalized Fisher's test by giving different d_i _df for each study. The test statistic under the null hypothesis has a $\chi^{2}\left(df=\overset{k}{\underset{i=1}{\sum }}d_{i}\right)$. More specifically, the test statistic is given by:

(2) $$\overset{K}{\underset{i=1}{\sum }}F_{i}^{-1}\left(1-p_{i}\right),$$

where $F_{i}^{-1}$ is the inverse of $\chi^{2}\left(df=d_{i}\right)$.

Rather than the chi-square distribution, a more generalized distribution, gamma distribution, can be used. The test statistic based on the gamma distribution can be written as:

(3) $$T=\overset{K}{\underset{i=1}{\sum }}G_{\alpha_{i}\beta }^{-1}\left(1-p_{i}\right),$$

where $G_{\alpha_{i}\beta }^{-1}\left(.\right)$ is the inverse gamma distribution with shape parameter *α_i _*and scale parameter *β*. Due to the property of the gamma distribution, for constant shape parameter *α_i_*, T will have a gamma distribution with shape parameter equals to $\overset{K}{\underset{i=1}{\sum }}\alpha_{i}$ and scale parameter equals to *β*. When all *α_i _*= 1, T has an exponential distribution under the null hypothesis. When all *α_i _*= *v*/2 and *β *= 2, the null distribution of T is a chi-square distribution with df=vK. When *v *= 1, it is the Chen-Nadarajah test [14]; when *v *= 2, it is the Fisher test.

In this paper, we use *β *= 1 as the scale parameter which has no effect on power of the test T. For the shape parameter, we will use $\alpha_{i}=1/p_{i}$ for the i^th ^study. So the proposed test statistic is:

(4) $$T=\overset{K}{\underset{i=1}{\sum }}G_{1/p_{i}}^{-1}\left(1-p_{i}\right),$$

Notice that since the gamma distribution with shape parameter *α_i _*and scale parameter 1 has expected value *α_i_*, a small p-value of *p_i _*results in a large expected value. Therefore, the proposed test gives larger "weights" to smaller p-values. In addition, since *p_i _*is a random variable, the proposed test doesn't follow a gamma distribution any more. However, the p-value can be easily estimated by resampling method. Under the null hypothesis, *p_i _*is uniformly distributed between 0 and 1. For the given number of studies, K, we can generate K numbers from uniform distribution U(0,1) and then calculate the statistic t defined in (4). We repeat this step N times (say, N = 10^8^), then the null distribution of T can be approximated by those numbers and the p-value can be estimated by the proportion of the N values which are greater than the observed statistic.

## Results

### Simulation study

To assess the performance of the proposed test, we conduct a simulation study by comparing it with some existing methods, including the z-test (denoted by Z), weighted z-tests with weights equal to the sample size (Z_n) or the estimated standard error (Z_se), the Chen-Nadarajah (CN) method, the Fisher test (Fisher). In the simulation study, we assume there are K independent studies, where K = 2, 10, or 100. For each study, we simulate data from two normal distributions: $N\left(0,\sigma^{2}\right)$, and $N\left(\mu_{i},\sigma^{2}\right)$ with sample sizes n_1 _= n_2 _= n, respectively. Of the K studies, there are different numbers of studies that have none-zero effects (i.e.,$\mu_{i}\neq 0$ ), which may have different values among studies but their sum is a constant. We consider several conditions for allocating effect sizes among the K studies. We first consider sample sizes and variances are fixed. We then assume the sample size, or the variance, or both the sample size and the variance are randomly sampled from given distributions. For random sample size, we assume it follows a Poisson distribution, Poi(λ); for random variances, we assume the standard deviation follows a gamma distribution with shape parameter *α *and scale parameter *β*, gamma(*α, β*). A p-value from a two-sample t-test to compare two group means for each study is obtained and is used to combine those K studies. When K is small (i.e., 2, and 10), we consider situations where there are 1 to K studies having none-zero effects. For K = 100, we consider i (i = 1, 2,..., 10) studies having the same effect size while the remaining 100-i studies having zero effect. We choose significance level 0.05 in the simulation study and use 10^5 ^replicates to estimate the type I error rate and the power.

### Simulation results

**Figure 1:**
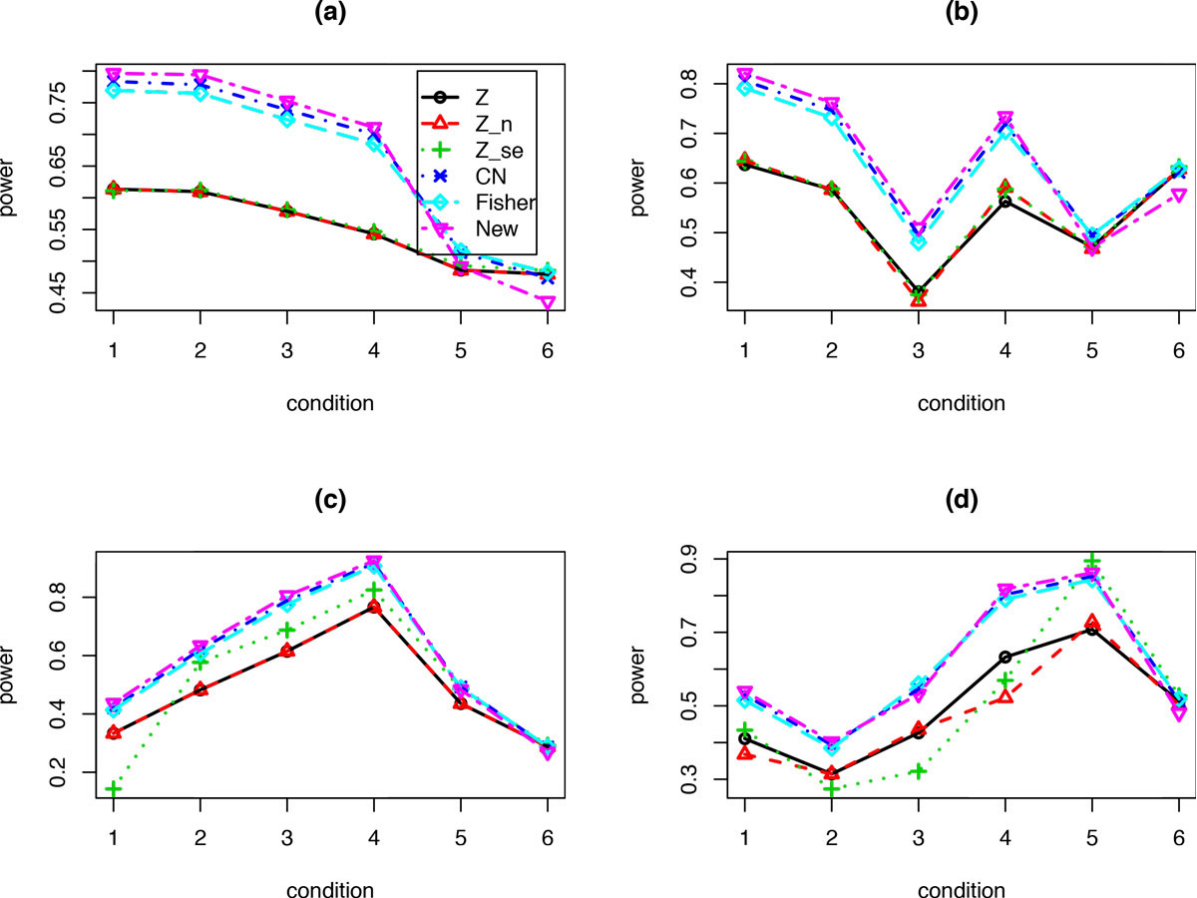
**Power values for each method under six different conditions: the total effect size is 1 and the ratios of the K = 2 effect sizes between study 1 and study 2 are 0, 0.01, 0.05, 0.1, 0.5, and 1**. (a) Sample sizes n = 20, and standard deviation σ = 1 for each study; (b) Same as (a), but the sample size n is a random sample from Poi(20); (c) Same as (a), but the standard deviation *σ *is a random sample from gamma(10, 0.1); (d) Same as (b), but the standard deviations *σ *is a random sample from gamma(10, 0.1).

All the methods can control type I error rate (data not shown). Figure 1 plots the power values for each method when there are only two studies (K = 2) with the sum of the two effect sizes equals to 1. Six conditions are considered: the ratios of the effects sizes between study1 and study 2 are 0, 0.01, 0.05, 0.1, 0.5, and 1, respectively. Therefore, the heterogeneity of effects between the two studies decreases from condition 1 to condition 6. Figure 1 (a) shows that the gamma-distribution based methods (Fisher, CN, and New) perform similarly, and all outperform the Z-based methods (Z, Z_n, Z_se) when the effects are not homogenous between the two studies (conditions 1 to 5). When the two studies have the same effect size (condition 6), the power values from all methods are close to each other. We have similar observations: when the sample size is randomly sampled from Poisson distribution Poi(20) (Figure 1 (b)), or when the standard deviation is randomly sampled from gamma distribution, gamma(10, 0.1) (Figure 1 (c)), or when both sample sizes and standard deviations are random samples as in Figure 1 (b) and 1(c) (Figure 1 (d)).

**Figure 2:**
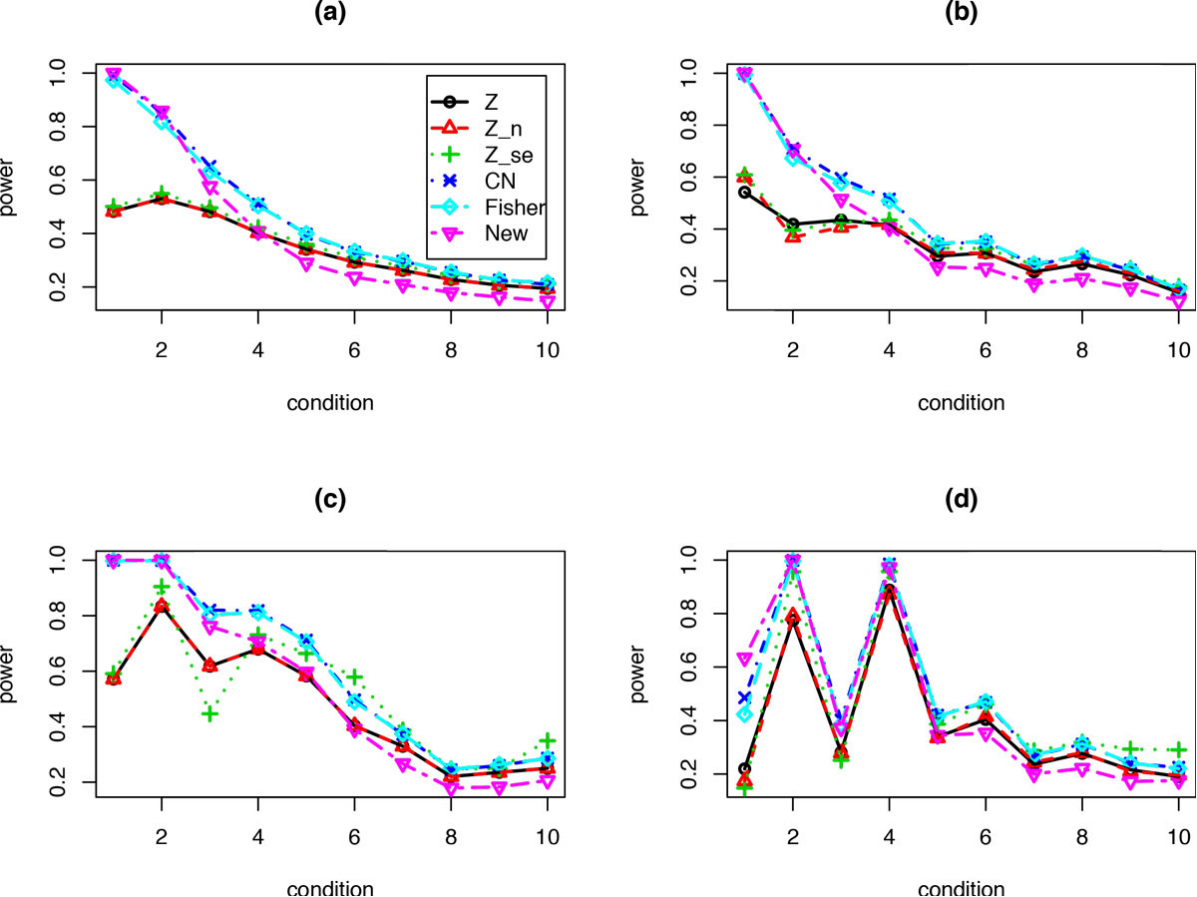
**Power values for each method under 10 different conditions: for situation i, there are i out of K = 10 studies each has the same effect size 2/i and the remaining 10-i studies have zero effect**. (a) Sample size n = 20, and standard deviation σ = 1 for each study; (b) Same as (a), but the sample size n is a random sample from Poi(20); (c) Same as (a), but the standard deviation *σ *is a random sample from gamma(10, 0.1); (d) Same as (b), but standard deviation *σ *is a random sample from gamma(10, 0.1).

Figure 2 plots the estimated power values for each method when there are i (i = 1,2,..., 10) studies having none-zero effects and the sum of those effect sizes is 2. For those i studies with none-zero effects, we assume the mean of the second group equals to 2/i. Figure 2 (a) shows that when only a few studies have none-zero effects (e.g., i = 1, 2, 3) the gamma-distribution based methods, including the proposed test perform better than those Z-based methods. However, when the effects among those studies become more homogenous, the proposed test has slightly lower power values. The sample size n = 20 and standard deviation σ = 1 are assumed for each study in Figure 2 (a). We have similar observations when sample size n is a random sample from Poi(20) (Figure 2 (b)), or when standard deviation *σ *is randomly sampled from a gamma(10, 0.1) (Figure 2 (c)), or when both sample size and standard deviation are random samples as in Figure 2 (b) and 2(c) (Figure 2 (d)).

**Figure 3:**
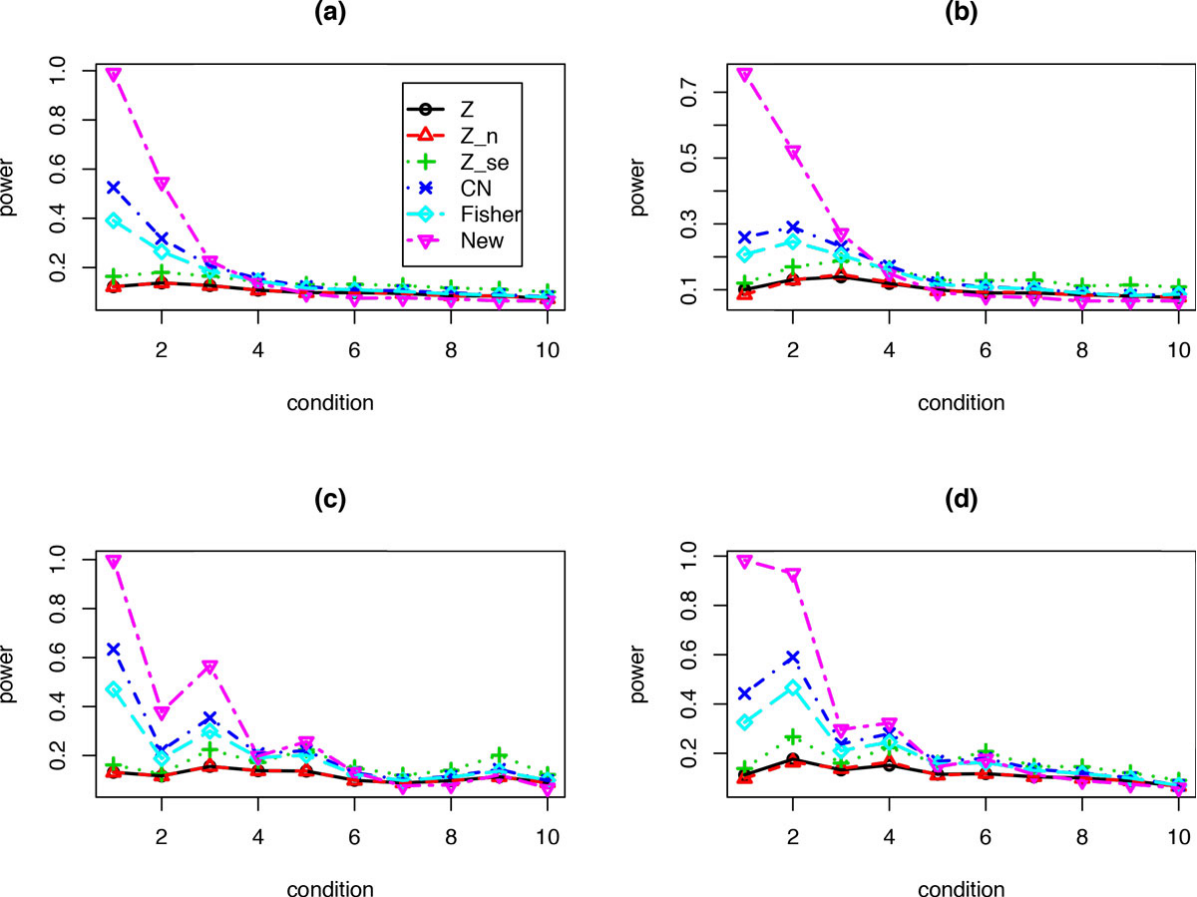
**Power values for each method under 10 different conditions: for situation i, there are i out of K = 100 studies each has the same effect size 2/i and the remaining 100-i studies have zero effect**. (a) Sample size n = 20, and standard deviation σ = 1 for each study; (b) Same as (a), but the sample size n is a random sample from Poi(20); (c) Same as (a), but the standard deviation *σ *is a random sample from gamma(10, 0.1); (d) Same as (b), but the standard deviation *σ *is a random sample from gamma(10, 0.1).

Figure 3 plots the power values for each method when there are 100 independent studies but only i(i = 1, 2,..., 10) studies have none-zero effect sizes. For those i studies with none-zero effects, we assume the mean for the second group equals to 2/i. In Figure 3 (a) we set sample size n = 20 and σ = 1 for each study. When there are only one or two studies having none-zero effects, the proposed test has much higher power values than the Fisher test and the CN method, which in turn are more powerful than the Z, Z_n, and Z_se tests. When the number of significant studies increases, all of the methods have close power values. The same pattern can be observed when n is random samples from Poi(20) (Figure 3 (b)), or when *σ *is randomly sampled from a gamma(10, 0.1) (Figure 3 (c)), or when both sample size and standard deviation are random samples as in Figure 3 (b) and 3(c) (Figure 3 (d)).

### Real data application

We apply the proposed approach to a meta-analysis of Genome-wide association study (GWAS). The data include 5 independent case-control studies where we wanted to test whether there is an association between the SNP rs17110747-A and major depression [28]. Table 1 is the count data from these studies. The 5 p-values are 0.94, 0.0015, 0.97, 0.79, and 0.81. The overall p-values from the Z, Z_n, Z_se, Fisher, CN, and the proposed test are 0.84, 0.53, 0.29, 0.17, 0.062, and 0.0081, respectively. Only the proposed test has p-value less than 0.05.

**Table 1:** Count data from the five independent studies investigating the association between SNP rs17110747-A and major depression.

study	case		control	
	
	event	total	event	total
1	11	270	25	630

2	244	1016	282	926

3	49	234	35	166

4	79	600	76	600

5	71	290	86	340

### Discussion and conclusions

Combining information from individual studies is an important and useful tool, especially for set-based approaches. For example, in studying the effect of rare variants on diseases, a set of rare variants are tested simultaneously, and their p-values are combined to test for the association between the rare variants and the disease [29]. However, most of the rare variants may have no or little effects while a few of them may have large effects. In this case, the proposed test will be more powerful than other methods if combining p-value methods are used. However, it should be pointed out that, the rare variants from a set (e.g., gene) maybe correlated, and the proposed test needs to be modified accordingly. A permutation-based test can be applied to estimate the p-value. We first calculate the statistic based on the proposed test (4). Then we permute the disease status (case or control); for each permutation, we use the proposed test to calculate a statistic. After a large number of permutations, the p-value will be estimated as the proportion of the statistics from the permutations excessing the observed statistic from the original data. To assess the performance of the proposed test in rare variant association studies, real data are needed. This will be a topic of our future research.

As mentioned earlier, no method is uniformly most powerful when combing p-values. However, based on our simulation studies, the proposed test is more powerful when the effects among the studies are more heterogeneous. When the effects are homogeneous, perhaps the Z-based tests are more powerful. Without the information about the effect sizes, robust methods, such as the CN and Fisher tests are recommended.

## Competing interests

The authors declare that they have no competing interests.

## Authors' contributions

ZC and MQY conceived and guided the project. ZC designed the project and conducted the study. WY, QL, JL, and JYY participated in the study through computational implementation, data analysis and discussions. ZC wrote the manuscript, and ZC and MQY finalized the manuscript, which was read and approved by all authors.

## Declaration

The funding for publication of the article has come from Indiana University Bloomington institutional research grant awards to ZC.

This article has been published as part of *BMC Bioinformatics *Volume 15 Supplement 17, 2014: Selected articles from the 2014 International Conference on Bioinformatics and Computational Biology. The full contents of the supplement are available online at http://www.biomedcentral.com/bmcbioinformatics/supplements/15/S17.
